# Mycotic Pseudoaneurysms of the Superior Mesenteric and Splenic Artery: A Case Report

**DOI:** 10.1155/crvm/5841946

**Published:** 2025-11-14

**Authors:** Fred Rudensky, Nausheen Merchant, Prasad Chalasani

**Affiliations:** ^1^Internal Medicine Graduate Medical Education Program, HCA Florida Lawnwood Hospital, Fort Pierce, Florida, USA; ^2^Florida State University College of Medicine, Fort Pierce Campus, Fort Pierce, Florida, USA; ^3^Cardiology, Florida State University College of Medicine, Tallahassee, Florida, USA

**Keywords:** endocarditis, mycotic pseudoaneurysm, splenic artery pseudoaneurysm, superior mesenteric artery pseudoaneurysm, visceral artery aneurysms

## Abstract

Visceral artery aneurysms and pseudoaneurysms are defined as aneurysms of the splenic, superior mesenteric, or inferior mesenteric arteries and their respective branches. Mycotic aneurysms, defined as aneurysms of the arterial wall caused by bacterial or fungal embolization, are a rapidly progressive and often fatal form of arterial aneurysms that can form in the visceral arteries. Aneurysms and pseudoaneurysms of the visceral arteries most commonly present as abdominal pain. The ambiguity with which they often present, paired with their high risk of rupture and hemorrhage, creates a highly precarious situation for clinicians. Failure to identify an aneurysm or pseudoaneurysm of the visceral arteries in time can be a fatal mistake. We present a case of mycotic pseudoaneurysms of the superior mesenteric artery and splenic artery secondary to infective endocarditis managed with open surgical resection, coil embolization, and splenectomy in a 36-year-old male with a history of intravenous drug use. The patient presented with a chief complaint of abdominal pain and confusion. He was admitted for sepsis and work-up of suspected bacteremia and endocarditis. MRI revealed multiple cortical infarcts suggestive of an embolic shower, and transesophageal echocardiogram showed mitral valve vegetations. CT imaging showed an aneurysm of the superior mesenteric artery, later determined to be a pseudoaneurysm. The patient underwent emergent open superior mesenteric artery pseudoaneurysm resection as well as splenectomy due to intraparenchymal pseudoaneurysms and associated necrosis and intraparenchymal hemorrhage. Our case highlights the importance of considering visceral artery aneurysms when formulating a list of differential diagnoses for patients presenting with abdominal pain due to their vague presenting symptoms in conjunction with their potential to rapidly progress to aneurysmal rupture and catastrophic hemorrhage.

## 1. Introduction

The estimated incidence of visceral artery aneurysm (VAA), including pseudoaneurysm, ranges from 0.01% to 0.2% [1–3], and the most commonly reported symptom is acute abdominal pain [4, 5]. Hepatic, splenic, and renal arteries are the most frequent sites of aneurysm, and the majority of these aneurysms are associated with atherosclerosis [1, 2]. The superior mesenteric artery (SMA) is the site of aneurysm in only 3.5%–8.0% of VAA cases [6, 7] and is most commonly caused by infection or dissection [1]. The Society of Vascular Surgery (SVS) guidelines for treatment of VAAs vary based on anatomical location, the presence of symptoms, and the size of the respective aneurysm. In contrast to other VAAs, the SVS recommends all aneurysms of the SMA be treated with surgical or endovascular repair [5–7]. We present a case of mycotic SMA pseudoaneurysm and multiple splenic intraparenchymal pseudoaneurysms secondary to left-sided methicillin-sensitive *Staphylococcus aureus* (MSSA) endocarditis and extensive thromboembolic shower in a young male with a history of intravenous drug use.

## 2. Case Presentation

The patient is a 36-year-old male with a past medical history of intravenous drug use admitted for severe sepsis secondary to suspected bacteremia. He reported 2 weeks of intermittent fever, confusion, headache, nausea, and vomiting. Noncontrast magnetic resonance imaging (MRI) of the brain utilizing multiple sequences was performed shortly after and showed numerous subacute cortical infarcts in the frontal, temporal, parietal, and occipital lobes, with the largest infarct measuring 2.5 cm, suggestive of an embolic shower. Computed tomography angiogram (CTA) of the chest utilizing routine standardized protocols with multiplanar reformation and 3D reconstruction was performed and revealed splenic enlargement with heterogenous perfusion raising suspicion for arterial phase ischemia or embolism. The patient underwent transthoracic echocardiogram (TTE) which did not show evidence of endocarditis. Blood cultures grew MSSA. A transesophageal echocardiogram (TEE) was ordered due to continued concern for endocarditis despite previously negative TTE. The TEE revealed a large vegetative growth involving both leaflets of the mitral valve and associated severe mitral regurgitation with near complete destruction of the mitral leaflets. Cardiothoracic surgery was consulted and recommended postponing surgical valve replacement for at least 1 month due to the patient's multiple subacute cortical infarctions.

On the tenth day of his hospital admission, the patient elected to leave against medical advice due to personal obligations. The following day, the patient returned to the emergency department with a chief complaint of severe centralized abdominal pain which he described as sharp and stabbing and was rapidly readmitted. The patient was found to be lethargic and confused on physical examination, with significant epigastric and periumbilical tenderness and guarding. Tachycardia and hypertension were noted on evaluation of vital signs (Table 1). Laboratory analysis revealed an elevated white blood cell count, erythrocyte sedimentation rate, and C-reactive protein, as well as multiple other abnormal laboratory values (Table 2).

**Figure 1:**
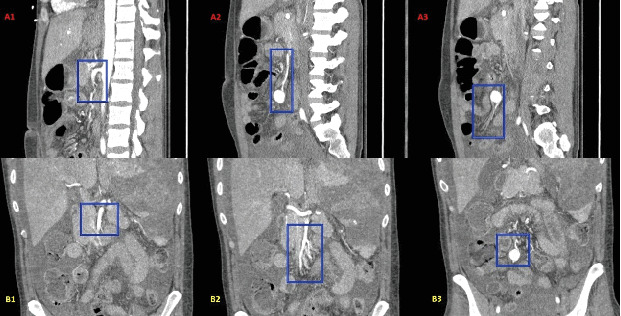
CTA of the abdomen and pelvis showing the course of the superior mesenteric artery and pseudoaneurysm in the sagittal and coronal views. (a1) Sagittal view of the abdomen with a blue rectangle outlining the origin of the superior mesenteric artery and adjacent abdominal aorta. (a2) Sagittal view of the abdomen with a blue rectangle outlining the proximal superior mesenteric artery and pseudoaneurysm. (a3) Sagittal view of the abdomen with a blue rectangle outlining the superior mesenteric artery pseudoaneurysm and distal arterial branches. (b1) Coronal view of the abdomen with a blue rectangle outlining the origin of the superior mesenteric artery and adjacent abdominal aorta. (b2) Coronal view of the abdomen with a blue rectangle outlining the proximal superior mesenteric artery and pseudoaneurysm. (b3) Coronal view of the abdomen with a blue rectangle outlining the superior mesenteric artery pseudoaneurysm and distal arterial branches.

The patient's known endocarditis with large mitral valve vegetations and multiple subacute cortical infarcts suggestive of an embolic shower, positive blood cultures, and the sudden onset of severe centralized abdominal pain raised suspicion for a symptomatic mycotic aneurysm. CTA of the abdomen and pelvis utilizing routine protocols with images taken in noncontrast, arterial, and venous phases confirmed the presence of an aneurysm arising from a distal branch of the SMA (Figure 1). Additional imaging findings included splenic intraparenchymal pseudoaneurysms, splenic necrosis and hemorrhage, and bilateral renal infarcts.

Emergent open aneurysm resection was performed. A pulsatile 3-cm mass consistent with mycotic pseudoaneurysm was resected from a distal branch of the SMA located in the area of the distal ileum and sent to pathology. In addition, a 5-cm defective segment of mesentery was resected, and the surrounding mesentery was approximated.

**Figure 2:**
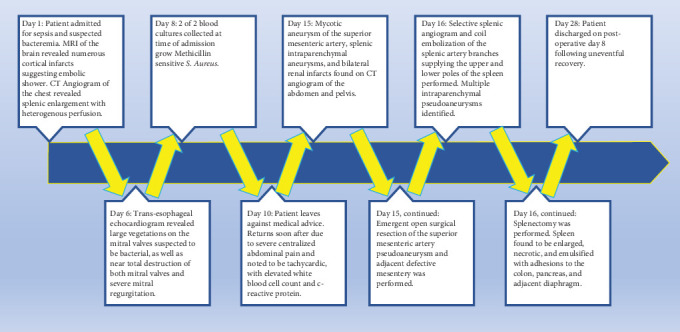
Timeline outlining the patient's hospital course from day of admission to day of discharge.

Removal of the enlarged spleen was planned for the following day due to concern for hemorrhage. Selective splenic artery angiogram and coil embolization of the splenic artery branches supplying the upper and lower poles of the spleen revealed two intraparenchymal pseudoaneurysms in the upper pole of the spleen as well as three tandem intraparenchymal pseudoaneurysms in the lower pole corresponding to the abnormalities noted on a previous CT angiogram of the abdomen and pelvis. The majority of the spleen was determined to be devascularized following coil embolization. Visual inspection during splenectomy revealed an enlarged, necrotic, and emulsified spleen with adhesions to the colon, pancreas, and diaphragm. The patient made a gradual recovery and was discharged after completion of the SMA pseudoaneurysm and defective segment of mesentery, coil embolization of the splenic artery branches, and splenectomy. Pathology reports for the tissue biopsies collected during surgical SMA resection and splenectomy were received shortly after the patient's discharge and definitively confirmed that the patient's pseudoaneurysm and splenic pseudoaneurysms were mycotic in origin. A chronological timeline of the patient's hospital course is provided in Figure 2.

## 3. Discussion

The management of the patient's SMA pseudoaneurysm required careful consideration at each step of treatment, and adherence to the SVS guidelines on the management of VAAs was maintained whenever possible. The SVS recommends an endovascular-first approach when treating aneurysms of the SMA due to significantly less morbidity in comparison to the surgical approach [5]. The patient's SMA pseudoaneurysm was treated with an open surgical approach as opposed to initially attempting endovascular repair due to the distal location of the patient's pseudoaneurysm. Endovascular repair of SMA aneurysms located beyond the proximal segment, such as in our case, requires sacrifice of major tributaries leading to significant morbidity [5]. Although the SVS recommends an endovascular-first approach for the treatment of SMA aneurysms, some studies, including a systematic review and meta-analysis comparing open to endovascular approaches in the treatment of all VAAs, have found no statistically significant difference in mortality between open and endovascular approaches [3, 8].

The SVS recommends treatment of all SMA aneurysms and pseudoaneurysms regardless of their respective characteristics. Although recent studies report a slower rate of aneurysm growth and rupture than previously documented [9], a 10-year retrospective study of 233 patients found no significant difference in diameter between ruptured and nonruptured VAAs [9, 10]. It is important to note that the diameter of several ruptured aneurysms could not be delineated. The study also reported a significant difference in risk of rupture between true aneurysms and pseudoaneurysms, with rupture occurring in only 3.1% of true aneurysms and 76.3% of pseudoaneurysms, respectively [9, 10].

Alternative methods of management, such as antibiotic therapy and close observation, were not pursued in our case. SMA aneurysms are most commonly mycotic [11]; however, more recent studies have reported degenerative aneurysms to be most commonly seen [12–14]. Mycotic aneurysms are seen more frequently in younger patients [7]. Mycotic aneurysms of the SMA are most often caused by infectious endocarditis, especially in cases of nonhemolytic streptococcus [7]. In the case of our patient, the presence of bacterial endocarditis, although caused by MSSA infection, the patient's young age, and the patient's aneurysm being located in the SMA significantly raised the likelihood of the identified VAA being mycotic in nature; 38%–50% of patients with aneurysm of the SMA present with already ruptured aneurysm [10, 14]. Mortality rates range from 30% to as high as 90% [10], and therefore, surgical intervention was determined to be necessary to prevent fatal complications stemming from possible aneurysmal rupture.

Certain limitations are noted in terms of management of the patient's VAAs and the reporting of our case. SMA aneurysms are unique in that 70%–90% of cases are symptomatic at the time of presentation. It is not possible to accurately determine the time of onset of the pseudoaneurysms, nor the rate of their progression. Since discharge, the patient has been lost to follow-up. Patients undergoing urgent splenectomy, such as in our case, should be vaccinated against *Streptococcus pneumoniae*, *Haemophilus influenzae* type B, and *Neisseria meningitidis* on or after Postoperative Day 14 [2, 15]. It is unclear if the patient received vaccination following hospital discharge. In addition, annual CTA scans are specific and sensitive for surveillance of VAA repairs [2]. It is also unclear whether the patient has undergone repeat CTA imaging following hospital discharge. Although our report provides a real-world example of the presentation, diagnosis, and treatment of mycotic pseudoaneurysms of the SMA and splenic artery, it is a single case.

## 4. Conclusions

Our case highlights the ambiguous presentation with which VAAs may present, as well as the emergent nature of visceral pseudoaneurysms. Although it is not possible to determine the time of onset of the mycotic SMA pseudoaneurysm, the patient had signs of systemic infection and abnormalities in the appearance of the spleen on CT imaging of the chest at least 10 days prior to the diagnosis of the pseudoaneurysm, raising the possibility that the SMA pseudoaneurysm was already developing. Earlier evaluation for intra-abdominal pathology may have allowed for a more timely diagnosis and treatment, preventing progression of the severity of our patient's condition. Our case highlights the importance of considering VAAs when formulating a list of differential diagnoses for patients presenting with abdominal pain due to their precarious combination of vague presentation, emergent nature, and high mortality rate. Our case emphasizes the need for a high index of suspicion for visceral artery pseudoaneurysms and consideration for early computed tomography imaging in patients with a history of intravenous drug use, endocarditis, evidence of embolic phenomena, unexplained sepsis, or persistent abdominal pain.

## Figures and Tables

**Table 1:** Vital signs at time of hospital readmission.

**Vital signs (units)**	**Value**
Temperature (°C)	36.9
Pulse (/minute)	106
Respiratory rate (/minute)	18
Blood pressure (mmHg)	161/96
SpO2 (%)	99

**Table 2:** Abnormal lab values at time of hospital readmission.

**Laboratory analysis (units)**	**Value**	**Reference value**
White blood cells (10^9^/L)	17.6	4–10.5
Hematocrit (%)	35.3	40.1–51
Hemoglobin (g/dL)	11.3	13.7–17.5
Platelets (10^9^/L)	669	150–400
Segmented neutrophils (%)	78	34–67.9
Sodium (mmol/L)	132	135–145
Total bilirubin (mg/dL)	1.9	0.1–1.2
Aspartate aminotransferase (U/L)	75	10–40
Troponin I (ng/dL)	0.074	0–0.034
C-reactive protein (mg/dL)	5	0–1
Erythrocyte sedimentation rate (mm/h)	57	0–15
Prothrombin time (s)	13.4	10–12.8
International normalized ratio	1.2	0.8–1.1

## Data Availability

The data that support the findings of this study are available from the corresponding author upon reasonable request.

## Supporting Information

Supporting Information Additional supporting information can be found online in the Supporting Information section. A copy of the 2013 CARE guidelines checklist for writing case reports has been completed and submitted as supporting information. This case report was written in adherence to the 2013 CARE guidelines. The majority of the requirements set forth in the guidelines were met.
