# Supplementary material for: Mycotic Pseudoaneurysms of the Superior Mesenteric and Splenic Artery: A Case Report
Source: Case Rep Vasc Med. 2025 Nov 14;2025:5841946. doi: 10.1155/crvm/5841946 (PMC12638144; doi:10.1155/crvm/5841946)
Supplement: Supporting Information — Additional supporting information can be found online in the Supporting Information section. A copy of the 2013 CARE guidelines checklist for writing case reports has been completed and submitted as supporting information. This case report was written in adherence to the 2013 CARE guidelines. The majority of the requirements set forth in the guidelines were met. [file 5841946.f1.pdf]

CARE Checklist of information to include when writing a case report

CARE case report guidelines

(cc) BY-NC-ND

Topic	Item	Checklist item description	Reported on Line
Title	1	The diagnosis or intervention of primary focus followed by the words "case report"	1,2
Key Words	2	2 to 5 key words that identify diagnoses or interventions in this case report, including "case report"	8,9
Abstract (no references)	3a	Introduction: What is unique about this case and what does it add to the scientific literature?	32-35
	3b	Main symptoms and/or important clinical findings	24-29
	3c	The main diagnoses, therapeutic interventions, and outcomes	29-31
	3d	Conclusion—What is the main "take-away" lesson(s) from this case?	32-35
Introduction	4	One or two paragraphs summarizing why this case is unique (**may include references**)	41-46
Patient Information	5a	De-identified patient specific information.	46-49, 51, 52
	5b	Primary concerns and symptoms of the patient.	52, 53
	5c	Medical, family, and psycho-social history including relevant genetic information	51, 52
	5d	Relevant past interventions with outcomes	NA
Clinical Findings	6	Describe significant physical examination (PE) and important clinical findings.	73-76, 79
Timeline	7	Historical and current information from this episode of care organized as a timeline	129
Diagnostic Assessment	8a	Diagnostic testing (such as PE, laboratory testing, imaging, surveys).	75-84
	8b	Diagnostic challenges (such as access to testing, financial, or cultural)	NA
	8c	Diagnosis (including other diagnoses considered)	88-92, 124-127
	8d	Prognosis (such as staging in oncology) where applicable	NA
Therapeutic Intervention	9a	Types of therapeutic intervention (such as pharmacologic, surgical, preventive, self-care)	109-120
	9b	Administration of therapeutic intervention (such as dosage, strength, duration)	NA
	9c	Changes in therapeutic intervention (with rationale)	NA
Follow-up and Outcomes	10a	Clinician and patient-assessed outcomes (if available)	121-124
	10b	Important follow-up diagnostic and other test results	NA
	10c	Intervention adherence and tolerability (How was this assessed?)	NA
	10d	Adverse and unanticipated events	70,71
Discussion	11a	A scientific discussion of the strengths AND limitations associated with this case report	133-181
	11b	Discussion of the relevant medical literature **with references**.	133-181
	11c	The scientific rationale for any conclusions (including assessment of possible causes)	169-172
	11d	The primary "take-away" lessons of this case report (without references) in a one paragraph conclusion	183-197
Patient Perspective	12	The patient should share their perspective in one to two paragraphs on the treatment(s) they received	NA
Informed Consent	13	Did the patient give informed consent? Please provide if requested	Yes ☒ No ☐
